# Supplementary material for: Outdoor sports and active tourism company management in Cordoba (southern Spain): An empirical study on the perception and behavior of supply
Source: PLoS One. 2020 Dec 11;15(12):e0243623. doi: 10.1371/journal.pone.0243623 (PMC7732107; doi:10.1371/journal.pone.0243623)
Supplement: S1 File — (DOCX) [file pone.0243623.s001.docx]

Survey for the analysis of active tourism supply and demand in the province of Cordoba, Spain

**Protocol**

I am conducting a research study on the social reality of physical adventure activities in nature in Cordoba and am interested in analyzing companies located in the province of Cordoba that offer various leisure activities related to these practices. We kindly request your collaboration in this rigorous scientific study on the supply and demand of these activities in Cordoba, the regulations and legislation related to this business sector, user trends, channels and access routes, contact with nature, the role of the media and future prospects.

Thank you for agreeing to take part in this ambitious project that aims to publicize these activities, which are growing in number and popularity in a society that increasingly demands leisure adventure tourism. In order for the information from this research to be the most helpful, it is important that you try to be as accurate, complete, and honest as possible with your answers.

The information you provide in the questionnaire will be kept in the strictest confidentiality. Once we have analyzed the data, a report will be delivered to the Tourism Delegation of Cordoba for dissemination to all the companies in the sector that have participated in the survey or will be published in a national journal.

Thank you for your participation

**1. Characteristics of the company**

- 1. Year registered in the Registry of Active Tourism Entities:

**______________________________________________________________________________**

- 1. Number of self-employed and employed workers:

**______________________________________________________________________________**

- 1. Legal form of the company:

**______________________________________________________________________________**

- 1. Company owner:

☐ Male

☐ Female

- 1. Age of company owner:

☐ 18–25 years ☐26–35 years

☐ 36–45 years ☐46–55 years

☐ 56 years and above

- 1. Available company resources:

☐ Human

☐ Material

☐ Infrastructure, equipment and tools

1.7. Location of company:

**______________________________________________________________________________**

1.8. Sites where activities are offered:

☐ Where the company is located ☐ Region of Andalusia

☐ Province of Cordoba ☐ Nationally

☐ Internationally ☐ Protected natural areas

1.9. Company’s initial registered capital:

☐ €0–€3,000 ☐ €3,001–€10,000

☐ €10,001–€20,000 ☐ €20,001–€30,000

☐ €30,001–€40,000 ☐ €40,001–€50,000

☐ €50,000–€60,000 ☐ More than €60,000

- 1. Net worth of the company in 2016:

☐ €0–€3,000 ☐ €3,001–€10,000

☐ €10,001–€20,000 ☐ €20,001–€30,000

☐ €30,001–€40,000 ☐ €40,001–€50,000

☐ €50,000–€60,000 ☐ More than €60,000

- 1. What was the turnover in the last 5 years?

YEAR 2012

☐ €0–€30,000 ☐ €30,001–€60,000

☐ €60,001–€90,000 ☐ €90,001–€120,000

☐ €120,001–€200,000 ☐ €200,001–€400,000

☐ €400,001–€600,000 ☐ More than €600,000

YEAR 2013

☐ €0–€30,000 ☐ €30,001–€60,000

☐ €60,001–€90,000 ☐ €90,001–€120,000

☐ €120,001–€200,000 ☐ €200,001–€400,000

☐ €400,001–€600,000 ☐ More than €600,000

YEAR 2014

☐ €0–€30,000 ☐ €30,001–€60,000

☐ €60,001–€90,000 ☐ €90,001–€120,000

☐ €120,001–€200,000 ☐ €200,001–€400,000

☐ €400,001–€600,000 ☐ More than €600,000

YEAR 2015

☐ €0–€30,000 ☐ €30,001–€60,000

☐ €60,001–€90,000 ☐ €90,001–€120,000

☐ €120,001–€200,000 ☐ €200,001–€400,000

☐ €400,001–€600,000 ☐ More than €600,000

YEAR 2016

☐ €0–€30,000 ☐ €30,001–€60,000

☐ €60,001–€90,000 ☐ €90,001–€120,000

☐ €120,001–€200,000 ☐ €200,001–€400,000

☐ €400,001–€600,000 ☐ More than €600,000

- 1. What were the company profits/ losses in these last 5 years?

YEAR 2012: Profit _________________ Loss____________________

YEAR 2013: Profit _________________ Loss____________________

YEAR 2014: Profit _________________Loss____________________

YEAR 2015: Profit _________________ Loss____________________

YEAR 2016: Profit _________________ Loss____________________

1.13. What insurance coverage do you provide your clients?

**______________________________________________________________________________**

1.14. Which of the following qualifications do the instructors of your company hold?

☐ Bachelor Degree in Physical Education

☐ Sports technician in a specific sports modality (Royal Decree 1913/1997 of 19 December).

☐ Higher sports technician in a specific sports modality (Royal Decree 1913/1997 of 19 December).

☐ Technician in physical and sports activities in the natural environment (Decree 190/1996 of 2 August).

☐ Technician in physical and sports activity animation (Decree 380/1996 of 2 August).

☐ Teacher, specialized in physical education (Royal Decree 1440/1911 of 30 August).

☐ Diploma in Physical Education (Royal Decree 790/1981 of 24 April).

☐ Degree in Physical Education (Royal Decree 790/1981 of 24 April).

☐ Higher technician in tourism animation (Decree 246/2001 of 6 November).

1.15. What is your company’s business management model?

☐ **CONCENTRATED–INTEGRATED.** Your active tourism company has joined an association or group to meet the needs of users in a region.

☐ **NON-CONCENTRATED–INDIVIDUALIZED.** Your company operates in isolation and carries out its activities independently without belonging to any type of association. You can work in a specific area or be very mobile.

☐ **NON-CONCENTRATED–INTEGRATED.** Your company forms part of an association or group but does not operate in a specific region.

1. **Information about supply**

2.1. What activities does your company currently offer?

☐ Climbing ☐ Cycling ☐ Horse riding ☐ Bungee jumping ☐ Hiking

☐ Trekking ☐ Canyoning ☐ Paragliding ☐ Hang gliding

☐ Skydiving ☐ Rafting ☐ Kayaking ☐ Archery

☐ Others (please specify) ______________________________________________

2.2. What new activities do you plan to offer this season?

________________________________________________________________________________________

2.3. Which activities do you plan to cancel, or have you canceled?

_________________________________________________________________________________________

2.4. What additional services do you offer?

☐ Accommodation

☐ Catering service

☐ Sale of merchandise

☐ Photography

☐ Video shooting

☐ Equipment rental

☐ Others (please specify) ____________________________________

2.5. Please indicate the price range of the activities.

€0–€10. Activities: ________________________________

€10–€20. Activities: _______________________________

€20–€30. Activities: _______________________________

€30–€40. Activities: _______________________________

€40–€50. Activities: _______________________________

More than €50. Activities: __________________________________

**3. Information about demand**

3.1. Please indicate the three most highly demanded activities in your company.

☐ Climbing ☐ Cycling ☐ Horse riding ☐ Bungee jumping ☐ Hiking

☐ Trekking ☐ Canyoning ☐ Paragliding ☐ Hang gliding

☐ Skydiving ☐ Rafting ☐ Kayaking ☐ Archery

☐ Others (please specify) ______________________________________________

3.2. Please indicate the three months of highest demand for active tourism activities.

☐ January ☐ February ☐ March ☐ April ☐ May ☐ June

☐ July ☐ August ☐ September ☐ October ☐November ☐ December

3.3. Which age groups are the most frequent?

☐ 8–16 years old ☐ 17–25 years old

☐ 26–35 years old ☐ 26–45 years old

☐ 45–55 years old ☐ 55 years old and above

3.4. Who are the most frequent users by sex?

☐ Male

☐ Female

- 1. Main geographical origin of users:

☐ Local ☐Provincial

☐ Regional ☐ National

☐ International

- 1. Principal level of user expertise:

☐ They frequently repeat the same activity.

☐ Expert

☐ Beginner

☐ Novice

☐ Don`t know/No answer

- 1. Users’ main motivation for practicing physical and sports activities in nature:

☐ Direct contact and interactive enjoyment of nature and a sense of freedom.

☐ Competitiveness and social relations in group practices

☐ Pursuing feelings of adventure, strong emotions, and controlled risk

☐ Pleasure, hedonism

☐ Practicing sport and physical exercise in an active, fun, and entertaining way.

☐ Don’t know/No answer.

1. **Current problems**

4.1. Do you think these activities have an environmental impact?

☐ Yes, specific types of activities have a significant environmental impact (e.g., motor sports and other more invasive activities).

☐ Yes, the activities frequently have a significant environmental impact and damage natural areas.

☐ The impact rates are not concerning.

☐ The natural environment is not significantly impacted by the primary activities provided by the company.

☐ Don’t know/No answer

4.2. Do you think the regulations and legislation concerning the current and new physical adventure activities in nature are timely and effective?

☐ There are significant loopholes in all aspects relating to physical adventure activities in nature.

☐ The activities are not regulated, but aspects regarding users and nature are.

☐ The regional government includes some activities but not others in its regulations and management measures.

☐ The administrative demands are excessive.

☐ Don’t know/No answer

4.3. Have the number of users increased or decreased?

4.3.1. If the number of users has decreased, what do you think are the reasons for the drop in demand?

☐ Economic (cost of activities, accommodation, travel, etc.)

☐ Fear of risk

☐ Not familiar with the activities

☐ Feeling of lack of safety

☐ No colleagues or friends to practice with

☐ Potential users’ lack of information about the activities

☐ Others (please specify) ________________________________

- - 1. If the number of users has increased, what do you think are the reasons for the rise in demand?

☐ Desire to experience new sensations

☐ Attractiveness of materials, equipment and nature

☐ Contact with nature in a highly industrialized society

☐ Opportunity to practice other types of sports besides traditional ones

☐ Ecological motives and the search for freedom

☐ Opportunity to experience a controlled adventure

☐ Others (please specify) _________________________________

- 1. What type of accidents/injuries occurred in 2016?

☐ Minor

☐ Moderate (dislocations, fractures, etc.)

☐ Serious

☐ None ______________________________________

- 1. If an accident/injury did occur, what was/were the cause(s) or reason(s)?

☐ User recklessness

☐ Instructor error

☐ Problems with equipment

☐ Due to the natural environment

☐ Don’t know/No answer

☐ Others (please specify) ______________________________________

**5. Promotion and advertising of company products**

5.1. What media do you mainly use to promote your company?

☐ Travel agencies, central reservation offices, and conventional tourism intermediaries

☐ Tourism associations

☐ Tourist information offices and other tourism bodies

☐ Radio advertisements

☐ Television advertisements

☐ Press advertisements

☐ Ads, information, and advertising on the Internet and in social networks

☐ Ads and advertising in specialized magazines

☐ Conventional posters

☐ Printed information leaflets

☐ None

☐ Others (please specify) __________________________________________

5.2. What is the company’s most profitable activity?

5.3. What is the company’s least profitable activity?

5.4. In terms of profitability, which activity would be the most interesting to promote?

5.5. Is your company identified with any activity or image? Please specify.

**6. Future prospects**

6.1. The sector

6.1.1. Which activity do you think has the most future potential? And the least?

+ Land ………………………. - Land ………………………….

+ Water ………………………… - Water ……………………………

+ Air…………………………. - Air…………………………….

6.2. Sport practices

6.2.1. What is your opinion about the future of physical adventure activities in nature?

☐ Activities clearly on the rise

☐ Timid growth or stabilization in the short and medium term

☐ Uncertain or unknown future

☐ These practices had their golden age but are now on the decline.

☐ Very uneven trends depending on the type of activity

☐ Worrying decline in users and athletes

☐ They will disappear.

☐ The free practice of nature sports will be made official and regulated until they lose their original identity.

☐ Others (please specify) _______

Encuesta para el análisis de la oferta y la demanda de Turismo Activo en Córdoba

**Protocolo**

Dentro del proceso de investigación que estoy llevando a cabo sobre el estudio de la realidad social de las actividades físicas de aventura en la naturaleza en Córdoba, hemos llegado al marco del análisis de las empresas ubicadas en los diferentes puntos de la provincia de Córdoba que ofertan diversas actividades de ocio relacionadas con estas prácticas. Pedimos su valiosa colaboración con el objeto de establecer un estudio científico y riguroso sobre la oferta y la demanda de estas actividades en Córdoba; la regulación y legislación, las tendencias de los usuarios, lo canales o vías de acceso, el contacto con el medio ambiente, el papel de los medios de comunicación y las perspectivas de futuro que se pueden dibujar.

Agradecemos su colaboración en este ambicioso proyecto que pretende dar a conocer, propagar y ayudar a difundir estas actividades, cada vez más numerosas y demandadas dentro de una sociedad que orienta su tiempo de ocio la turismo de aventura. Rogamos que trate de responder lo más fielmente posible a nuestro cuestionario, sin la necesidad de sentirse coaccionado por pregunta alguna y con la plena libertad de contestar.

Finalmente, le queremos asegurar que los datos obtenidos en este cuestionario serán tratados siempre de forma confidencial, una vez analizados y valorados efectuaremos una memoria que será entregada la Delegación Turismo de córdoba, para que lo haga llegar a todas las empresas del sector que hayan participado en esta encuesta, o en su defecto serán publicadas en una revista de ámbito nacional.

Muchas gracias por su participación

1. **Características de la empresa.**
   1. Año de inscripción en el Registro de Entidades de Turismo Activo:

**______________________________________________________________________________**

- 1. Número de trabajadores autónomos y por cuenta ajena:

**______________________________________________________________________________**

- 1. Figura jurídica empresarial:

**______________________________________________________________________________**

- 1. Titular de la empresa:

Hombre

Mujer

- 1. Edad del titular de la empresa:

De 18 a 25 años. De 26 a 35 años.

De 36 a 45 años. De 46 a 55 años.

De 56 en adelante

- 1. Recursos con los que cuenta la empresa:

Humanos

Materiales.

Infraestructuras, equipos y utillaje.

- 1. Localidad donde se ubica la empresa:

**______________________________________________________________________________**

- 1. Territorios o parajes donde desarrolla sus actividades.

Localidad donde se ubica.  Comunidad Autónoma Andaluza.

Provincia de Córdoba.  En el territorio nacional.

A nivel internacional.  Espacio natural protegido.

- 1. Capital inicial escriturado por la empresa.

Entre 0 y 3.000 euros.  Entre 3.001 y 10.000 euros.

Entre 10.001 y 20.000 euros.  Entre 20.001 y 30.000 euros.

Entre 30.001 y 40.000 euros.  Entre 40.001 y 50.000 euros.

Entre 50.000 y 60.000 euros.  Más de 60.000 euros.

- 1. Patrimonio neto de la empresa en el año 2016.

Entre 0 y 3.000 euros.  Entre 3.001 y 10.000 euros.

Entre 10.001 y 20.000 euros.  Entre 20.001 y 30.000 euros.

Entre 30.001 y 40.000 euros.  Entre 40.001 y 50.000 euros.

Entre 50.000 y 60.000 euros.  Más de 60.000 euros.

- 1. ¿Cuál es la facturación en estos últimos 5 años?

AÑO 2012

Entre 0 y 30.000 euros.  Entre 30.001 y 60.000 euros.

Entre 60.001 y 90.000 euros.  Entre 90.001 y 120.000 euros.

Entre 120.001 y 200.000 euros.  Entre 200.001 y 400.000 euros.

Entre 400.001 y 600.000 euros.  Más de 600.000 euros.

AÑO 2013

Entre 0 y 30.000 euros.  Entre 30.001 y 60.000 euros.

Entre 60.001 y 90.000 euros.  Entre 90.001 y 120.000 euros.

Entre 120.001 y 200.000 euros.  Entre 200.001 y 400.000 euros.

Entre 400.001 y 600.000 euros.  Más de 600.000 euros.

AÑO 2014

Entre 0 y 30.000 euros.  Entre 30.001 y 60.000 euros.

Entre 60.001 y 90.000 euros.  Entre 90.001 y 120.000 euros.

Entre 120.001 y 200.000 euros.  Entre 200.001 y 400.000 euros.

Entre 400.001 y 600.000 euros.  Más de 600.000 euros.

AÑO 2015

Entre 0 y 30.000 euros.  Entre 30.001 y 60.000 euros.

Entre 60.001 y 90.000 euros.  Entre 90.001 y 120.000 euros.

Entre 120.001 y 200.000 euros.  Entre 200.001 y 400.000 euros.

Entre 400.001 y 600.000 euros.  Más de 600.000 euros.

AÑO 2016

Entre 0 y 30.000 euros.  Entre 30.001 y 60.000 euros.

Entre 60.001 y 90.000 euros.  Entre 90.001 y 120.000 euros.

Entre 120.001 y 200.000 euros.  Entre 200.001 y 400.000 euros.

Entre 400.001 y 600.000 euros.  Más de 600.000 euros.

- 1. ¿Cuáles son los beneficios/pérdidas en estos últimos 5 años?

AÑO 2012: Beneficio _________________ Pérdida____________________

AÑO 2013: Beneficio _________________ Pérdida____________________

AÑO 2014: Beneficio _________________ Pérdida____________________

AÑO 2015: Beneficio _________________ Pérdida____________________

AÑO 2016: Beneficio _________________ Pérdida____________________

1.13. ¿Qué seguros ofrecen a sus usuarios?

**______________________________________________________________________________**

1.14. ¿Cuál de las siguientes titulaciones ostentan los monitores de su empresa?

☐ Licenciado/a en Ciencias de la Actividad Física y del Deporte.

☐ Técnico/a deportivo en la modalidad deportiva de que se trate (Real Decreto 1913/1997, de 19 de diciembre).

☐ Técnico/a deportivo superior en la modalidad deportiva de que se trate (Real Decreto 1913/1997, de 19 de diciembre).

☐ Técnico/a en conducción de actividades físico-deportivas en el medio natural (Decreto 2049/1995, de 22 de diciembre).

☐ Técnico/a en animación de actividades físicas y deportivas (Decreto 380/1996, de 2 de agosto).

☐ Maestro/a, especialidad en Educación Física (Real Decreto 1440/1911, de 30 de agosto).

☐ Diplomado/a en Educación Física (Real Decreto 790/1981, de 24 de abril).

☐ Licenciado en Educación Física (Real Decreto 790/1981, de 24 de abril).

☐ Técnico/a superior en animación turística (Decreto 246/2001, de 6 de noviembre).

1.15. ¿Qué tipo de gestión empresarial está desarrollando su empresa?

**CONCENTRADA-INTEGRADA**. Su empresa de Turismo activo se ha unido una asociación o agrupación para cubrir las necesidades de los usuarios de una región.

**NO CONCENTRADA-INDIVIDUALIZADA**. Su empresa está trabajando de forma aislada y ejerce sus actividades de forma independiente sin pertenecer a ningún tipo de asociación. Puedes trabajar en una zona en concreto o tener mucha movilidad.

**NO CONCENTRADA-INTEGRADA**. Cuando su empresa está unida a una asociación o agrupación, pero no ejercen sus funciones en una región determinada.

1. **Datos sobre la oferta**

2.1. ¿Qué actividades oferta actualmente su empresa?

Escalada  Bicicleta  Rutas a caballo  Puenting  Senderismo

Barranquismo  Parapente  Ala delta  Caída libre

Travesía  Rafting  Piragüismo  Tiro con arco

Otras. Indicar cuales______________________________________________

2.2. ¿Qué actividades nuevas tiene previsto ofertar para esta temporada?

_______________________________________________________________________________

2.3. ¿Y cuáles tiene previsto cancelar o ha cancelado?

_______________________________________________________________________________

2.4. ¿Qué servicios complementarios ofrecen?

Alojamiento.

Restauración.

Venta de merchandinsing.

Fotografía.

Videos.

Alquiler de equipos.

Otros. Especifique cuales____________________________________

2.5. Indica la franja de precios en las que están incluidas las distintas actividades.

Entre 0 y 10 euros. Actividad/es: ________________________________

Entre 10 y 20 euros. Actividad/es: _______________________________

Entre 20 y 30 euros. Actividad/es: _______________________________

Entre 30 y 40 euros. Actividad/es: _______________________________

Entre 40 y 50 euros. Actividad/es: _______________________________

Más de 50 euros. Actividad/es: __________________________________

**3. Datos sobre la demanda**

3.1. ¿Señale cuáles son las tres actividades más demandadas en su empresa?

Escalada  Bicicleta  Rutas a caballo  Puenting  Senderismo

Barranquismo  Parapente  Ala delta  Caída libre

Travesía  Rafting  Piragüismo  Tiro con arco

Otras. Indicar cuales______________________________________________

3.2. Señale cuales son los tres meses con mayor demanda para la práctica de las actividades de turismo activo.

Enero  Febrero  Marzo  Abril  Mayo  Junio

Julio  Agosto.  Septiembre  Octubre. Noviembre.  Diciembre

3.3. ¿Cuáles son los grupos de edades más comunes?

De 8 a 16 años. De 17 a 25 años.

De 26 a 35 años. De 26 a 45 años.

De 45 a 55 años.  Más de 55 años.

- 1. ¿Cuál es el sexo más habitual de los usuarios de los deportes de la naturaleza?

Hombre.

Mujer.

- 1. Principal procedencia geográfica de los usuarios:

Local Provincial

Comunidad Autónoma Nacional

Internacional

- 1. Nivel principal de los usuarios que practican deportes de la naturaleza:

Repiten frecuentemente la misma actividad.

Expertos.

Primera vez que practican.

Iniciados en los deportes de aventura.

No sabe/no contesta.

- 1. Motivación principal de los usuarios de las actividades físico-deportivas de la naturaleza:

Contacto directo y disfrute interactivo con la naturaleza y sensación de libertad.

Competitividad y relaciones sociales en prácticas de grupo.

Buscar sensaciones de aventura, emociones fuertes y riesgo controlado.

Placer, hedonismo.

Hacer deporte y ejercicio físico de manera activa, divertida y entretenida.

No sabe/no contesta.

**4. Problemática actual**

4.1. ¿Cree que existe un impacto ambiental con la práctica de estas actividades?

SI, existe impacto ambiental significativo, pero dependiendo del tipo de actividad y en el caso particular de los deportes de motor y algunos otros más invasivos de la naturaleza.

Sí, suele existir un importante impacto ambiental y de degradación de las áreas naturales afectadas.

No se dan índices de impacto que puedan ser preocupantes.

No existe impacto medio ambiental significativo de las actividades que mayoritariamente se realizan en el medio natural.

No sabe/no contesta.

4.2. ¿Cree que existe una legislación oportuna y eficaz que regule todas las actividades físicas de aventura en la naturaleza y las que surgieron nuevas?

Hay un vacío legal importante en todos los ámbitos de las AFAN.

No están reguladas las actividades pero sí en cambio, lo referente a los usuarios y a la naturaleza.

La Junta de Andalucía contempla dentro de su regulación y administración, unas actividades y otras no.

Las exigencias que nos pone la Administración son en cualquier caso excesivas.

No sabe/no contesta.

4.3. ¿Se incrementaron o disminuyeron el número de usuarios?

4.3.1. Si disminuyó, ¿Cuál cree que es el motivo por el que no existe más demanda de estas actividades?

Económico (precio de las actividades, alojamiento, desplazamiento, etc.)

Miedo a la sensación de riesgo.

Desconocimiento de las actividades

Sensación de inseguridad.

Falta de compañía o amigos para practicarlas.

Falta de información del posible usuario potencial.

Otros. Especifique cuales________________________________

- - 1. Si cree que se incrementaron, ¿Cuál es la causa por la cual cree que estas prácticas están en aumento?

Búsqueda de nuevas sensaciones.

Atractivo de los materiales, equipos y naturaleza.

Encuentro con la naturaleza dentro de una sociedad industrializada en exceso.

Practicar otras modalidades de deportes fuera de las tradicionales.

Corriente ecológica y de búsqueda de la libertad.

Posibilidad de vivir una aventura controlada.

Otras. Especifique cuales_________________________________

- 1. ¿Qué tipo accidentes sucedieron en el año 2016?

Accidentes leves.

Accidentes de cierta importancia (luxaciones, fracturas, etc.)

Accidentes graves.

No hubo accidentes.

- 1. ¿En caso afirmativo en la respuesta anterior, cuál/es es/son la/s causa/s o el/los motivo/s del/los accidente/s?

Imprudencia de los usuarios.

Error del monitor.

Problemas en el material.

Consecuencias de la naturaleza.

No sabe/no contesta.

Otras. Especifique cuales ______________________________________

**5. Promoción y publicidad que ofrece la empresa de sus productos**

5.1. ¿Cuál es la principal medio de promoción de su empresa?

Agencias de viajes, centrales de reserva e intermediarios turísticos convencionales.

Asociaciones turísticas.

Oficina de turismo y otras entidades competentes en turismo.

Anuncios en emisoras de radio.

Anuncios en cadenas de televisión.

Anuncios en prensa.

Anuncios, información y publicidad en Internet y redes sociales.

Anuncios y publicidad en revistas especializadas.

Cartelería convencional.

Trípticos informativos impresos.

Ninguno.

Otros. Especifique cuales__________________________________________

5.2. ¿Cuál es la actividad que más beneficios le reporta?

5.3. ¿Cuál es la actividad que menos beneficios le reporta?

5.4. ¿Cuál es la actividad que más interesa promocionar?

5.5. ¿Se identifica con alguna actividad o imagen su empresa? ¿Cuál?

**6. Perspectivas de futuro**

6.1. Sobre el sector

6.1.1. ¿Cuál es la actividad que cree que tiene más futuro? ¿Y la que menos?

+ Tierra………………………. - Tierra………………………….

+ Agua………………………… - Agua……………………………

+ Aire…………………………. - Aire…………………………….

6.2. Sobre las prácticas

6.2.1. ¿Cuál cree que será el futuro de las actividades físicas de aventura en la naturaleza?

Actividades claramente en accenso en general.

Crecimiento tímido o estabilización a corto y medio plazo.

Incierto o indefinido.

Estas prácticas han tenido su época dorada, pero ahora tienden a retroceder.

Tendencias muy desiguales dependiendo del tipo de actividad.

Preocupante descenso de usuarios y practicantes.

Desaparición.

Las prácticas libres de deportes de naturaleza se irán oficializando y reglando hasta perder su identidad original.

Otras. Especifique cuales____________________________________
